# B-Vitamins and Choline in Human Milk Are Not Impacted by a Preconception Lipid-Based Nutrient Supplement, but Differ Among Three Low-to-Middle Income Settings—Findings From the Women First Trial

**DOI:** 10.3389/fnut.2021.750680

**Published:** 2021-12-23

**Authors:** Bridget E. Young, Jamie Westcott, Jennifer Kemp, Lindsay Allen, Daniela Hampel, Ana L. Garcés, Lester Figueroa, Shivaprasad S. Goudar, Sangappa M. Dhaded, Manjunath Somannavar, Sarah Saleem, Sumera Aziz Ali, K. Michael Hambidge, Nancy F. Krebs, The Women First Working Group, Veena Herekar

**Affiliations:** ^1^Department of Pediatrics Allergy and Immunology, University of Rochester School of Medicine and Dentistry, Rochester, NY, United States; ^2^Department of Pediatrics, Section of Nutrition, University of Colorado Anschutz Medical Campus, Aurora, CO, United States; ^3^United States Department of Agriculture/Agricultural Research Service, Western Human Nutrition Research Center, Davis, CA, United States; ^4^Department of Nutrition, University of California, Davis, Davis, CA, United States; ^5^Department of Maternal and Newborn Health, Institute of Nutrition in Central America and Panama (INCAP), Guatemala City, Guatemala; ^6^KLE Academy of Higher Education and Research's, Jawaharlal Nehru Medical College, Belagavi, India; ^7^Department of Community Health Sciences, Aga Khan University, Karachi, Pakistan

**Keywords:** lipid nutrient supplement (LNS), human milk, B vitamins, thiamin, vitamin B12, nutrition intervention, infant growth

## Abstract

**Introduction:** Optimal human milk (HM) B-vitamin concentrations remain undefined, especially in areas where undernutrition is prevalent. The impact of supplementation pre-conception through pregnancy on HM B-vitamin composition remains unknown.

**Methods:** Human milk (HM) was collected at 2-weeks postpartum from 200 women in Guatemala, India, and Pakistan (the Women First Trial). The women were randomized to start a lipid-based nutrient supplement before conception, at end of the first trimester, or not at all; intervention continued until delivery. HM concentrations of eight B-vitamins and choline were assessed *via* ultra-performance liquid chromatography-tandem mass spectrometry. Maternal diet was assessed in early pregnancy, and infant growth followed through 6 months post-delivery.

**Results:** Despite supplement exposure averaging 15.7 (pre-conception arm) and 6.0 months (prenatal arm), HM B-vitamins did not differ between arms, but site differences were evident. Guatemala had higher HM concentrations of vitamin B3 than Pakistan and India. Pakistan had higher HM concentrations of thiamin and vitamin B6 than India and Guatemala. Cohort average HM vitamin B2 (162 ± 79 μg/L) and B6 (31.8 ± 24.6 μg/L) fell below values defined as deficient in 81.5 and 85.5% of samples, potentially reflecting sampling procedures and timing. Maternal dietary intakes of only vitamin B6 and choline were associated with the corresponding concentrations in HM (*p* < 0.005). No HM B-vitamin concentrations were associated with infant growth.

**Conclusion:** Prenatal supplementation for at least 6 months had no impact on HM B-vitamin concentrations at 2-weeks postpartum. Results suggest that the adequacy of HM composition was generally maintained, with potential exceptions of vitamin B2 and B6.

## Introduction

Dietary water-soluble vitamin recommendations for infants are based on a limited number of small studies of concentrations of these vitamins in human milk (HM) expressed for healthy, full-term, exclusively breastfed infants (1). The concentrations of nutrients in HM used to set these recommendations are often the average concentration detected in small studies of healthy lactating women several months postpartum (2). These recommendations differ by country and organization (2).

It is often postulated that adequate micronutrient concentrations in HM are maintained despite maternal undernutrition (3). However, reports exist of suboptimal concentrations of micronutrients, including water-soluble vitamins, in HM that result in nutrient deficiency in the recipient infant (4). It has become increasingly clear that more robust and standardized studies are needed to define what constitutes acceptable concentration ranges to support optimal infant intake in the first 6 months of life and beyond (2).

The impact of maternal nutrient status and dietary intake on concentrations in HM vary by nutrient. For most of the water-soluble vitamins including those studied here [thiamin (B1), riboflavin (B2), niacin (B3), vitamins B6, B12, pantothenic acid (B5), biotin (B7), and choline] maternal dietary intake and/or supplementation does have an impact on concentrations in HM (5). The worldwide prevalence of deficiency of these nutrients is difficult to estimate and may be elevated in certain at-risk populations with limited dietary diversity and/or access to animal source foods (5).

Some studies have investigated the impact of maternal lipid-based nutrient supplementation (LNS) during lactation on HM composition. Various LNS trials of lactating women have been shown to increase HM riboflavin, thiamin, pyridoxal, and vitamin B12 with conflicting results regarding the impact on HM thiamin (6, 7). However, direct supplementation with thiamin or thiamin-fortified foods during lactation both increase HM thiamin (8, 9). Similar studies have yielded analogous results with choline (10).

No randomized trials have investigated the impact of maternal supplementation from pre-conception throughout pregnancy. In populations where maternal undernutrition is pervasive, such studies are necessary. This study presents secondary data from three of the four sites of the Women First (WF) randomized control trial that assigned women to a lipid-based multiple micronutrient daily supplement at least 3 months prior to conception until delivery, or after the first trimester of pregnancy until delivery, or no supplement (11, 12). The trial took place in low-to-middle income countries (**LMIC**) where maternal undernutrition is prevalent.

Our goal was to investigate whether a pre-conception and/or prenatal nutrition intervention resulted in differences in selected B-vitamin and choline concentrations in HM, along with potential differences by study site or maternal body mass index (BMI). In turn, we investigated whether the variation in the vitamin concentration of HM was associated with infant growth patterns in these sites where infant growth faltering is common.

## Materials and Methods

These data are from three sites that participated in the WF Preconception Maternal Nutrition Trial (**WF**). The project was approved by the Colorado Multiple Institutional Review Board, University of Colorado, and the local and/or national ethics committees for each of the four sites. Written informed consent was obtained from all participants. The study protocol is available online: https://www.ncbi.nlm.nih.gov/pmc/articles/PMC4000057/. The trial is registered at ClinicalTrials.gov: NCT01883193.

The design and methods for this randomized controlled trial and the primary outcome (infant length at birth) have been previously published (11, 12). Briefly, women were recruited and randomized prior to conception in Guatemala (Chimaltenango), India (Belagavi, North Karnataka), and Pakistan (Thatta, Sindh Province). Due to the absence of an adequate cold chain, no samples or data are available for this report from the fourth WF site in the Democratic Republic of the Congo. At enrollment, the BMI was measured by study personnel and the women were randomized to Arm 1 = a small quantity lipid-based multiple micronutrient daily supplements (Supplement 1) for at least 3 months prior to conception; Arm 2 = received the daily Supplement 1 beginning at 12–14 weeks pregnancy; Arm 3 = no study supplementation. Additionally, women with a BMI < 20 kg/m^2^ at enrollment or whose gestational weight gain was below Institute of Medicine recommendations (13) were provided an additional daily 300 kcal energy-protein supplement (Supplement 2). The nutrient content of each supplement is presented in Supplementary Table 1. As such the maternal BMI at enrollment was categorized as “Low weight” = pre-conception BMI ≤ 20 kg/m^2^ (and thus provided Supplement 2); “Normal weight” = pre-conception BMI between 20 and 24.9 kg/m^2^; “Overweight” = pre-conception BMI ≥ 25.0 kg/m^2^. The compliance to the supplementation was estimated by collecting empty supplement packets along with maternal self-report using a daily calendar. All study supplementation ceased at delivery.

Maternal diet was assessed *via* two guided 24-h dietary recalls during the first trimester in a randomly selected subset of women from Arms 1 and 2 (before starting supplementation for Arm 2) who had confirmed pregnancy (*n* = 150). The dietary recalls were conducted 2–4 weeks apart at ~12–13 weeks of pregnancy. The dietary data were analyzed using site-specific food nutrient composition databases incorporating cultural-specific foods and local recipes. These food nutrient composition databases yielded estimates of daily intake of macronutrients and 12 micronutrients. Of the B-vitamins assessed in HM, maternal dietary intake estimates were available for: thiamin, riboflavin, vitamin B6 and B12, and choline. Dietary intake was not assessed in Arm 3. To estimate total nutrient intakes, maternal self-report of supplement compliance (as a proportion) was multiplied by the nutrients included in the supplements and then added to the nutrients from the dietary intake. Dietary intake and diversity scores of the populations have been previously published (14).

Mid-feed HM samples were collected *via* hand expression at the 2-week postpartum visit, occurring at variable times throughout the day. In short, the mothers washed their hands and cleaned the breast with clean water and gauze prior to feeding. The infant was allowed to initiate a breastfeeding session. Approximately 2 min after the breastfeeding session began, the infant was removed from the breast, and the mother hand-expressed roughly 20 ml of breast milk into sterile sample collection containers. The infant was then returned to the breast. Mid-feed collection was chosen to avoid inadvertently collecting either foremilk or hindmilk while utilizing hand expression as the collection method.

The HM sample was transferred into four cryovials and frozen at −80°C until shipment on dry ice to the Pediatric Nutrition lab (University of Colorado). The samples were collected by the assessment teams who were blinded to group assignment. No HM samples were collected from the women in Arm 3 in India. The HM samples were not light protected during the initial collection, aliquot, and shipping process. Once in the University of Colorado lab, the samples were aliquoted into light-protecting tubes under reduced-light conditions. Subsequent analyses of B-vitamin concentrations were conducted under light-protecting conditions.

The infant weight and length were measured by blinded study personnel at birth, 1, 3, and 6 months as previously described (11). The weight-for-age (WAZ), length-for-age (LAZ), and weight-for-length (WLZ) Z-scores were calculated according to the WHO standards, utilizing SAS Analytics Software (SAS Institute, Cary NC) codes provided by the Centers for Disease Control (15). The individual z-scores from 1, 3, and 6 months were plotted and linear regression fitted. The slope of this regression was used as the growth outcome rate for WAZ, LAZ, and WLZ.

### Assessment of Vitamins in HM

Thiamin, riboflavin, flavin adenine dinucleotide (FAD), flavin mononucleotide (FMN), nicotinamide (NAM), nicotinamide dinucleotide (NAD), pyridoxal (PL), pyridoxine (PN), biotin, and pantothenic acid were analyzed using ultra performance liquid chromatography—tandem mass spectrometry (UPLC-MS/MS) after protein precipitation and fat removal based on a previously published report (16). Vitamin B12 in milk was measured using a competitive protein binding assay as previously described (17). Choline, phosphocholine (PCho), and glycerophosphocholine (GPCho) were assessed using UPLC-MS/MS (18). The total vitamin concentrations for vitamin B2, B3, B6, and choline were calculated as follows: B2 = [riboflavin + (FAD^*^0.479) + (FMN^*^0.825)]; B3 = [NAM + (NAD^*^0.184)]; B6 = [PL + (PN^*^0.988)]; total choline = [choline + (PCHo^*^0.566) + (GPCho^*^0.405)].

Since only free thiamin could be measured, an adjustment factor was used to estimate the total thiamin concentrations in the HM samples. Based on the published proportional contribution of free thiamin to total thiamin concentrations in HM at 2–6 weeks postpartum, an adjustment factor of 10.4× was applied to estimate the total thiamin (19). All subsequent reports of total thiamine are based on this adjustment factor.

### Statistical Analysis

Unless otherwise noted, results are reported as means ± SD.

Vitamin concentrations were considered extreme outliers if they are >4× inter-quartile range above the median value. These values were excluded from means and associations reported to be conservative. All relationships were tested including and excluding any outliers to ensure the nature of relationships remained the same.

Differences in maternal characteristics by site and arm were tested *via* ANOVA. Differences in milk components by maternal pre-conception BMI category, site, and arm were tested with ANOVA or Wilcoxon Rank Sum depending on the normality of the distribution of milk variable (tested *via* Shapiro-Wilk test). If significant differences were detected, Tukey's test was utilized to determine individual differences between sites and arms. Multivariable regression was used to test for differences in HM B-vitamin concentrations between maternal BMI categories, controlling for the site as a covariate.

The maternal dietary intake of riboflavin, vitamin B12, thiamin, and choline in early pregnancy including intake from supplements, accounting for self-reported supplement compliance, were tested for correlations with corresponding concentrations in HM using simple linear regression. Multivariable regression was used to test for relationships between maternal dietary intake and HM composition, controlling for the site.

Concentrations of vitamins in HM were also tested for association with infant growth outcomes using linear regression.

In all regression models, non-normal variables were transformed (using log or square root transformation) as necessary to ensure the normality of the models' residuals.

To control for multiple comparisons, *p* < 0.01 was considered significant.

Analyses were conducted using SAS 9.4 and JMP Pro 14 (SAS Institute, Cary NC).

## Results

### Cohort Characteristics

A total of 200 women were included: 75 from Guatemala (25 in each of the three arms); 50 from India (25 in Arm 1 and 2); 75 in Pakistan (25 in each arm). The maternal characteristics are presented in Table 1. The average length of time women received the supplement was 15.7 ± 3.5 months in Arm 1 and 6.0 ± 0.6 months in Arm 2.

**Table 1:** Maternal characteristics^1^.

**Variable**	**All sites**	**Guatemala**	**India**	**Pakistan**	***p*** ^**2**^
BMI at enrollment (kg/m^2^)	22.1 ± 4.6	25.8 ± 4.3^a^	19.4 ± 2.7^b^	20.2 ± 3.1^b^	<0.0001
Arm 1	21.8 ± 4.4	24.9 ± 4.4	19.5 ± 3.0	20.8 ± 3.8	
Arm 2	22.2 ± 4.6	26.9 ± 4.0	19.4 ± 2.5	20.2 ± 2.9	
Arm 3	22.6 ± 4.6	25.6 ± 4.4		19.7 ± 2.6	
BMI ≤ 20 kg/m^2^ at enrollment (%)	37.2%	4.0%^a^	63.3%^b^	53.3%^b^	<0.0001
Arm 1	37.8%	0%	62.5%	52.0%	
Arm 2	37.3%	4.0%	64.0%	44.0%	
Arm 3	36.0%	8.0%		64.0%	
Age at enrollment (years)	23.6 ± 3.9	23.9 ± 3.8^a^, ^b^	21.8 ± 2.8^a^	24.7 ± 4.4^b^	0.0004
Arm 1	22.8 ± 3.7	23.3 ± 3.8	21.5 ± 2.5	23.4 ± 4.5	
Arm 2	23.9 ± 3.8	23.7 ± 3.4	22.2 ± 2.8	25.8 ± 4.1	
Arm 3	24.7 ± 4.2	24.6 ± 4.1		24.8 ± 4.4	
Gestational age at delivery (weeks)	38.9 ± 1.7	39.0 ± 1.4^a^, ^b^	39.6 ± 1.6^a^	38.4 ± 1.8^b^	0.0006
Arm 1	38.7 ± 1.9	39.0 ± 1.4	39.5 ± 1.5	37.7 ± 2.1	
Arm 2	39.1 ± 1.6	39.1 ± 1.4	39.6 ± 1.8	38.7 ± 1.4	
Arm 3	39.0 ± 1.5	39.0 ± 1.4		38.9 ± 1.6	
Infant sex (male)	53%	51%	56%	53%	0.88
Arm 1	49%	44%	54%	48%	
Arm 2	55%	56%	56%	52%	
Arm 3	56%	52%		60%	
Milk collection time (days)	13.1 ± 1.2	13.1 ± 0.1	12.8 ± 0.2	13.4 ± 0.1	0.010
Arm 1	13.1 ± 1.3	12.8 ± 1.2	12.9 ± 0.8	13.6 ± 1.6	
Arm 2	13.2 ± 1.1	13.4 ± 1.5	12.9 ± 0.8	13.4 ± 0.7	
Arm 3	13.1 ± 1.0	12.9 ± 0.9		13.4 ± 1.2	

1 *Mean ± SD presented*.

2 *For differences by site*.

a, b, c *Different letter superscripts indicate significantly different means between sites, Tukey or “N−1” Chi-square test (20): p < 0.0003*.

### B-Vitamins in HM by Site and Arm, and Maternal BMI at Enrollment

Biotin concentrations were under the limit of detection for 14.5% of samples measured. Pantothenic acid concentrations were under the limit of detection for 0.5% of the samples measured. In the analyses, these samples were assigned the lowest detectable concentration.

**Figure 1:**
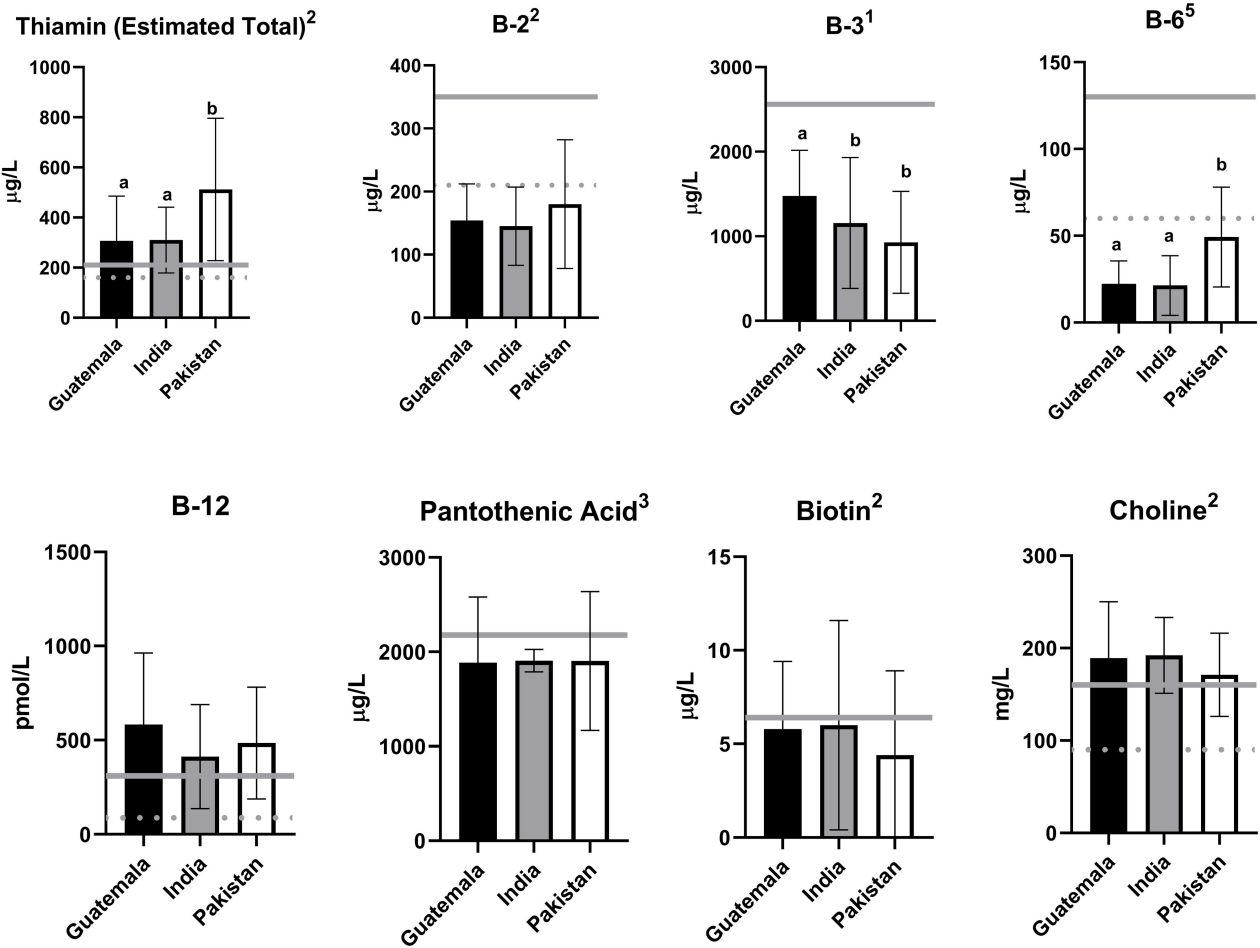
Human milk (HM) B-vitamin concentrations at 2-weeks postpartum by site. Shown are the mean ± SD of the concentration of human milk B-vitamins at 2-weeks. Solid gray lines represent the concentration in human milk used to set the Adequate Intake (AI) (21), the dietary reference intake for infants 0–6 months in the USA, assuming an intake of 780 ml per day. Dashed gray lines represent concentrations in human milk associated with a deficiency in the recipient exclusively breastfed infant for thiamin, vitamin B2, B6, B12, and choline, summarized in Allen et al. (4). Estimated total thiamin concentration based on free thiamin measurement and previously published adjustment factor at 2–6-week human milk (19). ^1^One outlier excluded from the reported mean; ^2^Two outliers excluded from the reported mean; ^3^Three outliers excluded from the reported mean; ^5^Five outliers excluded from the reported mean; Differences between sites were assessed *via* ANOVA and Wilcoxon Rank Sum Test in normally distributed and non-normally distributed variables, respectively. When *p* < 0.0001, significant differences between individual sites were assessed *via* Tukey's Test and are indicated by different letters.

There were no differences in the HM vitamin concentrations by study arm. There were also no differences in HM vitamin concentrations by study arm within individual sites (concentrations presented by site and arm in Supplementary Table 2). All HM vitamins assessed differed by site, except for vitamin B2, B12, and pantothenic acid. The mean concentrations for each site are depicted in Figure 1.

The HM vitamin B3 and biotin concentrations were different by maternal enrollment BMI. Total HM vitamin B3 was lower in mothers with “Low weight” (979 ± 680 μg/L, *n* = 75) compared with mothers with “normal” (1,277 ± 682 μg/L, *n* = 79) and “overweight” (1,490 ± 886 μg/L, *n* = 46) enrollment BMI (*p* = 0.0004). However, when controlling for site, this difference between BMI categories was no longer significant (parameter estimate for “Maternal BMI” in the model: *p* = 0.285). HM biotin was lowest in mothers with “Low weight” (4.6 ± 4.9 μg/L, *n* = 75) and highest in mothers with “overweight” (6.4 ± 3.9 μg/L, *n* = 46) enrollment BMI (*p* = 0.007). This difference remained significant when controlling for the site (parameter estimate for “Maternal BMI” in the model: *p* = 0.019).

There were no differences in HM B-vitamin concentrations between mothers delivering male vs. female infants.

### Prevalence of Concentrations Below Values Used to Set Dietary Recommendations

Vitamin concentrations in HM were compared with the concentrations used to set the US adequate intake (AI) for infants (which assumes 780 ml/day intake of HM). Vitamin concentrations in HM were also compared with previously published concentrations associated with deficiency based on clinical manifestations of deficiency in the recipient exclusively breastfed infant (4). There were no differences by study arm in the prevalence of HM below the concentration used to set the AI or below concentrations reported to be deficient.

The proportion of HM samples falling below these concentrations in each site individually is reported in Table 2, along with the expected temporal trend of concentrations in HM over the first few months of lactation. Of note, 81.5 and 85.5% of samples had concentrations of vitamin B2 and vitamin B6 that fell below concentrations previously associated with deficiency (4) (respectively) and 56.5% of samples had vitamin B3 concentrations less than one half of the concentration used to set the AI (21).

**Table 2:** Prevalence of human milk (HM) vitamin concentrations falling below concentrations used to set dietary guidelines.

**Nutrient**	**Change over time^a^**	**[Milk] for setting AI^b^**	**% Meeting AI**	**% <0.5 AI**	**[HM] reported deficiency^c^**	**% Deficient [HM]**	***p*** **^d^**
Estimated total thiamin^e^	↑	210 μg/L	All: 82%	All: 1.5%	160 μg/L	All: 6.5%	0.0013
			G: 69.3%	G: 2.7%		G: 14.7%	
			I: 86.0%	I: 2.0%		I: 2.0%	
			P: 92.0%	P: 0%		P: 1.3%	
Total B2	↓	350 μg/L	All: 4.0%	All: 67.5%	210 μg/L	All: 81.5%	0.13
			G: 1.3%	G: 69.3%		G: 82.7%	
			I: 2.0%	I: 78.0%		I: 90.0%	
			P: 8.0%	P: 58.7%		P: 74.7%	
Total B3	↑	2560 μg/L	All: 3.5%	All: 56.5%			0.0002
			G: 5.3%	G: 40.0%			
			I: 4.0%	I: 56.0%			
			P: 1.3%	P: 73.3%			
Total B6	↑	130 μg/L	All: 3.0%	All: 87.0%	60 μg/L	All: 85.5%	<0.0001
			G: 0%	G: 98.7%		G: 98.7%	
			I: 0%	I: 98.0%		I: 96.0%	
			P: 8.0%	P: 68.0%		P: 65.3%	
Total B12	↓	310 pmol/L	All: 59.5%	All: 2.0%	88.6 pmol/L	All: 0%	N/A
			G: 61.3%	G: 2.7%		G: 0%	
			I: 46.0%	I: 4.0%		I: 0%	
			P: 66.7%	P: 0%		P: 0%	
Pantothenic acid	–	2,179 μg/L	All: 31.5%	All: 9.5%			0.87
			G: 28.0%	G: 10.7%			
			I: 38%	I: 8.0%			
			P: 30.7%	P: 9.3%			
Biotin	–	6.41 μg/L	All: 35.5%	All: 39.5%			0.006
			G: 42.6%	G: 25.3%			
			I: 38.0%	I: 38.0%			
			P: 26.7%	P: 53.3%			
Total Choline	↑	160 mg/L	All: 74%	All: 2.5%	90 mg/L	All: 2.5%	0.37
			G: 78.7%	G: 4.0%		G: 4.0%	
			I: 84.0%	I: 0%		I: 0%	
			P: 62.7%	P: 2.7%		P: 2.7%	

*AI, adequate intake for infants 0–6 months; All, prevalence in all sites combined; G, Guatemala; I, India; P, Pakistan*.

a *Change over time from ~2-weeks to several months of lactation; summarized: (5, 22–24)*.

b *Assuming 780 ml/day*.

c *Summarized in Allen et al. (4)*.

d *p-value for Pearson comparison of distribution between sites (either % deficiency HM concentrations for thiamin, B12 and choline, or % of HM concentrations below 50% of the concentration used to set the AI for vitamin B3, pantothenic acid, and biotin)*.

e *Estimated total thiamin concentration based on free thiamin measurement and previously published adjustment factor at 2–6 weeks human milk (19)*.

### Association With Maternal Diet

Dietary intake data from early pregnancy was available from 150 women (50 from each site) who were randomized to arm 1 or 2. Maternal early pregnancy dietary intake (excluding and including supplementation) of thiamin, vitamin B2 (riboflavin), B6, B12, and choline are presented by the individual site in Table 3 alongside the estimated average requirement (EAR) for these nutrients during lactation.

**Table 3:** Maternal daily dietary intake of thiamin, vitamin B12, and choline in early pregnancy.

	**EAR^a^**	**Guatemala**	**India**	**Pakistan**	**All**	***p*** **^2^**
Thiamin—diet only	1.2 mg	1.4 ± 0.5^a^	0.5 ± 0.2^b^	1.2 ± 0.5^a^	1.1 ± 0.6	<0.0001
Thiamin—diet + supplement		3.3 ± 0.8^a^	3.1 ± 0.3^a^	3.7 ± 0.7^b^	3.4 ± 0.7	<0.0005
Riboflavin—diet only	1.3 mg	1.5 ± 0.4^a^	0.8 ± 0.3^b^	1.2 ± 0.6^c^	1.2 ± 0.6	<0.0001
Riboflavin—diet + supplement		3.4 ± 0.7	3.4 ± 0.1	3.6 ± 0.8	3.5 ± 0.7	0.22
Vitamin B6—diet only	1.7 mg	2.0 ± 0.7^a^	1.0 ± 0.4^b^	2.0 ± 1.0^a^	1.6 ± 1.9	<0.0001
Vitamin B6—diet + supplement		4.6 ± 1.0^a^	4.5 ± 0.5^a^	5.3 ± 1.2^b^	4.8 ± 1.0	0.0003
Vitamin B12—diet only ^3^	2.4 mg	1.7 ± 0.3^a^	0.9 ± 0.6^b^	1.8 ± 1.2^a^	1.4 ± 1.1	<0.0001
Vitamin B12—diet + supplement ^3^		5.3 ± 1.8^a^	5.6 ± 0.7^a^, ^b^	6.3 ± 0.2^b^	5.9 ± 2.1	0.0135
Choline—diet only^4^	550 mg	261 ± 123^a^	120 ± 41^b^	145 ± 73^b^	176 ± 105	<0.0001

*Maternal dietary intake in early pregnancy including all study supplements accounting for self-reported compliance to supplementation throughout the study was reported as Mean ± SD*.

*^1^EAR, estimated average requirement = The estimated daily intake necessary to meet the needs of 50% of healthy lactating women ages 19–30 years old in the USA*.

2 *Differences between sites were assessed via ANOVA*.

3 *One outlier excluded*.

4 *Choline was not included in the study supplements*.

a, b, c *Tukey's Test was used to assess pairwise differences between individual sites. Significant differences were indicated by different letters (p < 0.01)*.

The maternal dietary intake (including study supplementation) of these vitamins was compared with the corresponding vitamin concentrations in HM. In the bivariate analyses, the maternal intake of vitamin B12 (in a subset of women) was not associated with the HM concentrations of B12 (*p* = 0.09). Maternal intake of thiamin, riboflavin, vitamin B6, and choline tended to associate with corresponding concentrations of thiamin, vitamin B2, B6, and choline in HM, respectively (thiamin: *p* = 0.023, *R*^2^ = 0.04, *n* = 150; vitamin B2: *p* = 0.015, *R*^2^ = 0.04, *n* = 149; vitamin B6: *p* = 0.003, *R*^2^ = 0.06, *n* = 150; choline: *p* = 0.004, *R*^2^ = 0.06, *n* = 150). In the multivariable analyses of these relationships, controlling for site, the significance of maternal dietary intake of each vitamin (as estimated by parameter estimate *p* values) was reduced (Table 4).

**Table 4:** Multivariable model of the relationships between maternal dietary intake on HM B-vitamin concentrations, controlling for site.

**HM vitamin Concentrations**	**Predictors^a^**	**Model** ***p*** **value**	***Model R*** **^2^**	**Parameter** ***p*** **value**	***n***
Vitamin B2	Site	0.173	0.033	0.085	150
	Maternal dietary riboflavin			0.672	
Vitamin B6	Site	<0.0001	0.300	<0.0001	150
	Maternal dietary B6			0.325	
Thiamin	Site	<0.0001	0.189	<0.0001	150
	Maternal dietary thiamin			0.434	
Vitamin B12^b^	Site	0.099	0.042	0.060	149
	Maternal dietary B12			0.288	
Choline	Site	0.011	0.054	0.232	150
	Maternal dietary choline			0.015	

*HM, human milk*.

a *Maternal dietary intake includes intake of supplement accounting for compliance to supplementation. Supplement 1 did not include choline*.

b *1 outlier excluded*.

### Associations With Infant Growth

None of the vitamin concentrations in HM were significantly associated with WAZ, LAZ, or WLZ rate from 1 to 6 months (associations presented in Supplementary Table 3).

## Discussion

These are the first data to report the impact of a maternal small-quantity multiple micronutrient LNS from pre-conception through birth on B-vitamin concentrations in HM, making a significant contribution to the evidence base for global HM composition. The duration of supplementation was substantial, averaging 16 months before delivery (pre-conception arm) and 6 months before delivery (prenatal arm). Yet, supplementation had no demonstrable impact on vitamin concentrations at 2-weeks postpartum compared with women receiving no supplementation. HM composition significantly differed between these three low-to-middle income settings, likely reflecting underlying differences in maternal phenotype, habitual diet, and lifestyle. Lastly, the prevalence of HM concentrations that fell below values used to set breastfed infant intake recommendations for vitamin B2 and B6 warrants future investigation into the risk of infant deficiencies in these settings. These data reinforce the need for well-designed studies to define infant nutrient requirements during the period of exclusive breastfeeding (25).

It is interesting that several months of supplementation pre-conception with LNS did not impact HM composition despite providing the daily RDA of pantothenic acid and twice the RDA of thiamin, niacin, and vitamin B12. The supplementation intervention was completed at delivery, supporting previous assertions that HM concentrations of water-soluble vitamins are more affected by acute rather than historical maternal intake (26). Previous work has shown that maternal supplementation with water-soluble vitamins can be reflected in increased HM concentrations as soon as hours after consumption (27). Additionally, postpartum LNS begun at birth resulted in detectable increases in HM vitamin B2, B3, B6, and B12 (but not thiamin) concentrations as early as 2-weeks postpartum (7). These data together suggest that our sampling of HM at 2-weeks may have coincided with the waning of any impact of prenatal LNS supplementation on HM composition.

In these cohorts, there were far more differences in HM B-vitamins among sites than by intervention arm. HM from Guatemalan women tended to have higher concentrations of vitamin B12 (*p* = 0.05) and had significantly higher concentrations of vitamin B3 (*p* < 0.001). These differences could be attributed to differences in maternal dietary intake (14) and BMI (12) among sites, as controlling for differences in maternal vitamin B12 intake did explain a significant proportion of the variation in HM vitamin B12 concentrations. Pakistani mothers produced HM with higher concentrations of thiamin and vitamin B6, which may partially be attributed to differences in habitual dietary intake and consumption of animal source food (22, 28). We have previously shown that adequate dietary diversity was only reported in 20% of the full Pakistani cohort, 50% of the full Guatemalan cohort, and 70% of the full Indian cohort (14). We have also previously shown many of the Guatemalan mothers exhibited greater protein intake during pregnancy (1.04 g/kg/day) compared with the Pakistani (0.82 g/kg/day) and Indian (0.67 g/kg/day) mothers (14). Whether these underlying dietary pattern differences contributed to differences observed in HM composition at 2-weeks remains a topic of future research.

There are several important considerations for interpreting the vitamin concentrations reported here. First, the concentrations in HM for several of these vitamins change over time (particularly thiamin, vitamin B6, B12, and choline); the dynamics of this change are impacted by maternal BMI and dietary intake (28). Secondly, caution is warranted when comparing concentrations in our samples collected at 2-weeks with concentrations used to set the AI. While the AI is set for infants 0–6 months, the HM samples used to set these reference ranges are often collected several months into lactation in studies with small sample sizes. Lastly, thiamin, vitamins B2, B3, B6, B12, and biotin are all light-sensitive (16, 29). While our collection procedures are similar (or even more light-sparing) to other contemporary studies of B-vitamins in HM, concentrations reported herein may be underestimates of concentrations provided to the infant when nursing directly at the breast. It is useful to note that the HM thiamin and vitamin B12 concentrations we detected suggest that limited light-induced degradation occurred during initial sample processing.

The majority of milk samples exhibited vitamin B2 and B6 concentrations shown to be deficient in previous studies (4). As vitamin B2 concentrations in HM decrease or remain constant over time, the prevalence of milk considered “deficient” in vitamin B2 (81.5%) would likely remain high if samples were collected later in lactation. A smaller study of HM collected earlier at 2–8 days lactation in Brazil found vitamin B2 concentrations even lower, at 89 μg/L (28) (compared with our cohort mean of 162 μg/L). Suboptimal maternal and HM vitamin B2 concentrations are pervasive internationally and have resulted in reports of insufficient infant status (5). These results warrant future studies to investigate functional outcomes associated with a deficiency in these at-risk infants.

Vitamin B6 concentrations in HM increase over the first several weeks of lactation (23, 27, 30, 31). Thus, estimates of deficiency (85.5%) are likely to be inflated in this study due to our early sampling time point. In fact, the combined site mean of this study was 31.8 ± 24.6 μg/L, which is slightly higher than the mean of 416 samples collected from healthy women across 11 provinces in China between 8 and 14 days postpartum (26.4 ± 31.8; *p* = 0.037) (23). However, other studies of vitamin B6 in HM also sampled at 14 days report concentrations between 2 and 10 × higher than the average concentrations detected in these cohorts (30, 32). Poor vitamin B6 status in infants at 6 months has been associated with slower growth and behavioral outcomes (33, 34), and higher HM concentrations of B6 have been associated with higher neurobehavioral functioning assessment scores in the neonatal period (35).

A large proportion of samples exhibited vitamin B3 concentrations that were <50% of the concentration used to set the AI. However, our estimates of total vitamin B3 include measurements of nicotinamide and nicotinamide dinucleotide and are thus an underestimate of total HM B3 (which also contains nicotinamide dinucleotide phosphate, nicotinamide riboside, and nicotinamide mononucleotide). The global prevalence of vitamin B3 deficiency is unknown, but HM concentrations <20% of the concentrations used to set the AI have been documented in many populations from LMICs and industrialized countries (22), corroborating our detection of low concentrations. HM concentrations of vitamin B3 have been reported to both increase and remain constant after 2 weeks of lactation (22, 23, 28). Indeed, a smaller study of HM collected earlier, at 2–8 days lactation in a Brazilian cohort, found vitamin B3 concentrations even lower, at 544 μg/L (28) (compared with our cohort mean of 1,189 μg/L) suggesting an increase over time. Thus, the timing of our sampling may have contributed to a falsely high prevalence of HM falling <50% of the AI reference concentrations. It is of note that there were no relationships between any of the HM vitamin concentrations and measures of infant growth, indicating that infant intake of vitamins B2, B3, and B6 were sufficient to support relatively normal weight and linear growth patterns (36). Rigorous longitudinal data including assessment of functional outcomes of infant micronutrient deficiency are necessary to address the range of concentrations of these vitamins in HM that are optimal for infant growth and development (2).

The majority of HM concentrations of the estimated total thiamin were above the concentrations used to set the AI for these nutrients, and thiamin concentrations can be expected to increase over the first few months (5, 23, 24). This is interesting given that previous studies have documented concerningly low concentrations in HM thiamin in similar populations (8, 19). For example, our combined population estimated total HM thiamin of 384 ± 236 μg/L is higher than total thiamin detected in 335 lactating women at 2-weeks lactation (129 ± 75 μg/L, *p* < 0.0001) in Cambodia (9), an area where thiamin deficiency is prevalent (37). These results are reassuring that HM thiamin concentrations are likely meeting infant needs in these sites.

Previous studies have also documented concerningly low concentrations in HM vitamin B12 among similar populations. In fact, a study of lactating women in Guatemala City found that 65% of the milk samples collected at 12 months had undetectable vitamin B12 concentrations (38). However, we measured the HM at ~2-weeks postpartum, and vitamin B12 concentrations decrease over the first several weeks of lactation, stabilizing at 2–4 months (39), which may partially explain the higher average concentration we detected compared with other studies in similar populations that assessed milk later in lactation. These relatively higher concentrations of vitamin B12 in HM are reassuring that infant needs are likely being met in spite of the fact that >80% of the women in this trial exhibited inadequate dietary intake of vitamin B12 in early pregnancy (14).

HM biotin concentrations are stable over time. In these samples, 39.5% exhibited concentrations less than one-half that were used to set the biotin AI (6.41 μg/L). The cohort mean of biotin (5.3 ± 4.5 μg/L) is significantly lower than the mean of 113 HM samples from Indonesian mothers at 2-5.3 months postpartum (18.3 ± 1.6 μg/L; *p* < 0.0001) (40). These comparisons emphasize the need for large and rigorous studies to formalize reference ranges of such nutrients in HM.

Pantothenic acid in HM does not severely fluctuate over time and is not light sensitive. Roughly 1/3 of this study population had HM concentrations of pantothenic acid above those used to set the AI. Only 9.5% of HM samples had concentrations <50% of that used to set the AI. However, the cohort mean of HM pantothenic acid (1,897 ± 713 μg/L) is significantly lower than the mean of 416 samples collected between 9 and 14 days postpartum across 11 provinces in China (2,881 ± 1,605 μg/L; *p* < 0.0001) (23). In contrast, the mean concentration we observed was significantly higher than the mean of 113 HM samples from Indonesian mothers at 2–5.3 months postpartum (1,540 ± 871 μg/L; *p* < 0.0001) (40). The cause of the discrepancies in concentrations between these cohorts is unknown but may be attributable to differences in dietary intakes between cultures.

Choline concentrations increase from early transitional to mature milk (5, 23, 24). Choline in HM is not light-sensitive. In this study, 74% of the HM exhibited choline concentrations above those used to set the infant AI. In our population, the HM choline concentration was 183 ± 51 mg/L, which is higher than the HM choline concentrations assessed at 8 weeks in Canadian and Cambodian mothers (134 ± 36 mg/L; *p* < 0.0001) (41). This is of particular note given that the supplement in this trial was not fortified with choline indicating that the majority of women in our trial were able to adequately meet choline needs from diet alone.

Maternal dietary intake (including study supplementation) was inconsistently associated with HM concentrations by the nutrient in this cohort. We detected no relationship between the maternal dietary intake of vitamin B12 during the first trimester of pregnancy and HM concentrations at 2-weeks post partum. The relationship between maternal vitamin B12 status and HM concentrations is still not fully understood, but there is some evidence that HM concentrations are correlated with maternal habitual intake (39) and bolus supplementation (6). The lack of association between maternal vitamin B12 intake and HM concentrations may be because maternal dietary intake during the first trimester of pregnancy may not be a good reflection of maternal dietary intake at the time of milk collection when supplementation was withdrawn. There was a trend for a positive relationship between the maternal intake of thiamin, riboflavin, and vitamin B6 and HM concentrations, but these relationships were weakened once controlled for the site. Others have shown that increased maternal intake of thiamin-fortified food results in increased HM thiamin concentrations (8), but our dietary intake and HM collection were separated by ~30 weeks. The supplement provided twice the RDA for thiamin, riboflavin, vitamin B6, and B12. However, HM concentrations did not differ by study arm, so any impact of supplementation on HM composition was minimal at best, even after many months of supplementation exposure. Maternal choline intake remained a predictor of HM choline concentrations even when controlling for the site. The supplement in this trial was not fortified with choline. In controlled feeding trials, mothers with high choline intakes (930 vs. 480 mg/day; both higher than average intake in this cohort) exhibited higher HM choline concentrations (10). This corroborates our data that show that HM choline concentrations are indeed related to maternal intake in early pregnancy. However, the low correlations coefficients of these relationships (*R*^2^ <0.07) make the clinical significance uncertain.

The B-vitamin concentrations in HM are impacted by maternal acute supplementation/intake (42), but this would not be expected to differ by intervention arm as supplementation was withdrawn at birth. This study did not control for time of day at collection. However, a recent review and meta-analysis found no evidence for circadian variation in any of the B-vitamins assessed in HM (43).

This study has many strengths. This is the first randomized controlled trial to supplement women at risk for undernutrition from pre-conception and early gestation through pregnancy with a small-quantity lipid-based supplement. The inclusion of a control group of women from the same communities is a significant strength. The sample size is quite large for studies including longitudinal assessment of infant growth and biochemical assessment of HM samples. It also included women from three distinct locations and very diverse dietary patterns, allowing for insight into geographical factors impacting variability in HM outcomes. Finally, the inclusion of maternal dietary intake data is a strength that many other investigations of HM composition lack. These results contribute to the extremely limited evidence base for expected B-vitamin concentrations in HM in a globally diverse population.

Certain weaknesses limit the interpretation of results. Maternal dietary intake was not assessed at the time of milk collection. Supplementation was withdrawn at birth, and thus, we cannot investigate if continued supplementation may have impacted HM composition. We reported HM measurements at one time point, relatively early in the post-partum period (2-weeks), which complicates interpretation as concentrations of water-soluble vitamins are dynamic in the early weeks post-partum. We did not assess any functional outcomes in the infant beyond growth (WAZ, LAZ, and WLZ), and are thus, unable to report any symptoms of nutrient deficiency. Lastly, milk volume was not measured, so infant nutrient intakes can only be estimated, though extent of breastfeeding during the early weeks postpartum was high in all of the sites (36).

## Conclusion

In conclusion, these data reflect that maternal long-term LNS supplementation from pre-conception to birth does not impact water-soluble vitamin concentrations in HM measured at 2-weeks postpartum. The adequate early postnatal growth of infants provides reassurance of the quality of HM to support growth in these settings. However, the prevalence of HM samples with vitamin B2, B3, and B6 concentrations below current reference concentrations in these limited data warrants further longitudinal studies and functional investigations in infants. With no demonstrable benefit of maternal supplementation for at least 6 months before lactation, the concentrations of other B-vitamins in these women suggest that the adequacy of milk composition was generally maintained. These results contribute to the evidence base for HM composition and affirm the need for rigorous data to effectively define optimal intakes of these B-vitamins in infants and corresponding concentrations in HM throughout lactation.

## Data Availability Statement

The datasets presented in this article are not readily available because request for data should be sent to the senior author. Requests to access the datasets should be directed to nancy.krebs@cuanschutz.edu.

## Ethics Statement

The studies involving human participants were reviewed and approved by the Colorado Multiple Institutional Review Board and local Ethics Committees at each site. Written informed consent from the participants' legal guardian/next of kin was not required to participate in this study in accordance with the national legislation and the institutional requirements.

## The Women First Working Group

Veena Herekar: Academy of Higher Education and Research, Belagavi, Karnataka, India. Sunil S. Vernekar: Academy of Higher Education and Research, Belagavi, Karnataka, India. S. Yogeshkumar: Academy of Higher Education and Research, Belagavi, Karnataka, India. Umber Khan: Aga Khan University, Karachi, Pakistan. Farina Abrejo: Aga Khan University, Karachi, Pakistan. Robert K. Goldenberg: Columbia University Medical Center. Richard J. Derman: Thomas Jefferson University. Beth McClure: RTI International. Marion Koso-Thomas: NIH/NICHD.

## Author Contributions

BY, KH, and NK contributed to the study conception and design. JK, DH, AG, LF, SG, SD, MS, SS, and SA contributed to the data collection. BY, JW, JK, DH, LA, KH, and NK contributed to the analysis and interpretation of results. BY, JW, KH, and NK contributed to the manuscript preparation. All authors contributed to substantial revision to and approved the final version of the manuscript.

## Funding

Supported by the Bill and Melinda Gates Foundation, Seattle, WA (OPP1055867) and the National Institutes of Health *Eunice Kennedy Shriver* NICHD and the Office of Dietary Supplements U10 HD 076474 and UG1 HD 076474.

## Conflict of Interest

The authors declare that the research was conducted in the absence of any commercial or financial relationships that could be construed as a potential conflict of interest.

## Publisher's Note

All claims expressed in this article are solely those of the authors and do not necessarily represent those of their affiliated organizations, or those of the publisher, the editors and the reviewers. Any product that may be evaluated in this article, or claim that may be made by its manufacturer, is not guaranteed or endorsed by the publisher.
