# Supplementary material for: B-Vitamins and Choline in Human Milk Are Not Impacted by a Preconception Lipid-Based Nutrient Supplement, but Differ Among Three Low-to-Middle Income Settings—Findings From the Women First Trial
Source: Front Nutr. 2021 Dec 23;8:750680. doi: 10.3389/fnut.2021.750680 (PMC8733746; doi:10.3389/fnut.2021.750680)
Supplement: Supplementary file 1 [file Table_1.docx]

Supplementary Material

***Supplemental Table 1.*** Nutritional specifications for Supplement 1 and Supplement 2 given to maternal participants of the Women First Maternal Preconception Trial (5)

| **Nutrient^1^** | **Supplement 1: 20 g sachet^2^** | **Supplement 2:**  **55 g sachet^2^** |
| --- | --- | --- |
| Energy, *kcal* | 118 | 302 |
| Protein, *g* | 2.6 | 11.2 |
| Lipid, *g* | 10 | 20.6 |
| Linoleic Acid^3^, *g* | 4.5 | 5.65 |
| α-Linolenic Acid^3^, *g* | 0.5 | 0.44 |
| Calcium^3^, *mg* | 280 | 104 |
| Phosphorus (total) ^3^, *mg*  Phosphorus (free) ^3^, *mg* | 190  *NA* | 174  106 |
| Potassium^3^, *mg* | 200 | 563 |
| Magnesium^3^, *mg* | 65 | 78 |
| Zinc^3^, *mg* | 15 | 1.2 |
| Copper^3^, *mg* | 4 | 0.24 |
| Iron^3^, *mg* | 20 | 1.9 |
| Folate, *μg* | 400 | *NA* |
| Iodine, *μg* | 250 | *NA* |
| Manganese, *mg* | 2.6 | *NA* |
| Niacin, *mg* | 36 | *NA* |
| Pantothenic Acid, *mg* | 7 | *NA* |
| Riboflavin, *mg* | 2.8 | *NA* |
| Selenium, *μg* | 130 | *NA* |
| Thiamine (B1), *mg* | 2.8 | *NA* |
| Vitamin A, *μg* | 800 | *NA* |
| Vitamin B12, *μg* | 5.2 | *NA* |
| Vitamin B6, *mg* | 3.8 | *NA* |
| Vitamin C, *mg* | 100 | *NA* |
| Vitamin D2, *IU* | 1000 | *NA* |
| Vitamin E, *mg* | 20 | *NA* |
| Vitamin K, *μg* | 45 | *NA* |

^1^Data provided by Nutriset (Malauney, France)

^2^Recommended daily dose; Supplement 1 given on daily basis to women in Arm 1 for at least 3 months prior to conception and to women in Arm 2 starting late in the first trimester. Supplement 2 was given to anyone on Supplement 1 who had a BMI ≤ 20 or with inadequate weight gain during pregnancy. Both supplements were discontinued at delivery.

^3^Nutrient content provided by raw materials.

**Supplementary Table 2: Human milk vitamin concentrations by site and intervention arm**

| **Supplementary Table 2.** | | | | | | |
| --- | --- | --- | --- | --- | --- | --- |
|  | **Arm 1** | | **Arm 2** | | **Arm 3** | |
|  | Mean ± SD | n | Mean ± SD | n | Mean ± SD | n |
| **Estimated Total Thiamin µg/L (2 outliers excluded)** | | | | | | |
| Guatemala | 303 ± 146 | 25 | 300 ± 129 | 25 | 316 ± 242 | 25 |
| India | 313 ± 100 | 24 | 307 ± 158 | 24 | --- |  |
| Pakistan | 564 ± 299 | 25 | 521 ± 299 | 25 | 454 ± 253 | 25 |
| By Arm^1^ | 391 ± 230 | 74 | 377 ± 232 | 74 | 385 ± 255 | 50 |
| **Total Riboflavin (Vitamin B2) µg/L (2 outliers excluded)** | | | | | | |
| Guatemala | 146 ± 50 | 25 | 168 ± 70 | 24 | 148 ± 52 | 25 |
| India | 142 ± 57 | 25 | 148 ± 68 | 25 |  |  |
| Pakistan | 197 ± 99 | 25 | 182 ± 118 | 25 | 161 ± 85 | 24 |
| By Arm | 162 ± 75 | 75 | 166 ± 89 | 74 | 155 ± 70 | 49 |
| **Total Niacin (Vitamin B3) µg/L (1 outlier excluded)** | | | | | | |
| Guatemala | 1475 ± 563 | 24 | 1572 ± 518 | 25 | 1378 ± 549 | 25 |
| India | 1122 ± 626 | 25 | 1193 ± 911 | 25 | --- |  |
| Pakistan | 845 ± 577 | 25 | 1056 ± 614 | 25 | 883 ± 619 | 25 |
| By Arm | 1143 ± 636 | 74 | 1274 ± 726 | 75 | 1131 ± 631 | 50 |
| **Total B6 µg/L (5 outliers excluded)** | | | | | | |
| Guatemala | 22.0 ± 13.3 | 25 | 21.5 ± 12.4 | 25 | 23.5 ± 14.5 | 25 |
| India | 22.2 ± 14.8 | 25 | 20.6 ± 19.6 | 25 |  |  |
| Pakistan | 53.4 ± 25.8 | 24 | 42.1 ± 25.9 | 22 | 51.9 ± 33.4 | 24 |
| By Arm | 32.2 ± 23.6 | 74 | 27.5 ± 21.8 | 72 | 37.4 ± 29.0 | 49 |
| **Total B12 pmol/L** | | | | | | |
| Guatemala | 597 ± 388 | 25 | 538 ± 371 | 25 | 615 ± 392 | 25 |
| India | 421 ± 289 | 25 | 405 ± 268 | 25 | --- |  |
| Pakistan | 499 ± 352 | 25 | 523 ± 306 | 25 | 432 ± 224 | 25 |
| By Arm | 505 ± 349 | 75 | 489 ± 319 | 75 | 524 ± 329 | 50 |
| **Pantothenic Acid µg/L (3 outliers excluded)** | | | | | | |
| Guatemala | 2012 ± 796 | 25 | 1727 ± 627 | 24 | 1909 ± 645 | 25 |
| India | 1944 ± 686 | 24 | 1869 ± 763 | 24 | --- |  |
| Pakistan | 1786 ± 914 | 25 | 1982 ± 603 | 25 | 1942 ± 667 | 25 |
| By Arm | 1914 ± 800 | 74 | 1861 ± 666 | 73 | 1926 ± 650 | 50 |
| **Biotin (Vitamin B7) µg/L (2 outliers excluded)** | | | | | | |
| Guatemala | 6.4 ± 4.0 | 25 | 6.1 ± 3.7 | 25 | 4.9 ± 3.0 | 25 |
| India | 7.1 ± 5.7 | 24 | 4.8 ± 5.5 | 24 | --- |  |
| Pakistan | 3.8 ± 4.4 | 25 | 5.5 ± 5.2 | 25 | 4.0 ± 3.8 | 25 |
| By Arm | 5.7 ± 4.9 | 74 | 5.5 ± 4.8 | 74 | 4.4 ± 3.4 | 50 |
| **Total Choline mg/L (2 outliers excluded)** | | | | | | |
| Guatemala | 193 ± 69 | 24 | 185 ± 66 | 24 | 190 ± 48 | 25 |
| India | 192 ± 39 | 25 | 192 ± 44 | 25 | --- |  |
| Pakistan | 169 ± 50 | 25 | 177 ± 46 | 25 | 166 ± 39 | 25 |
| By Arm | 185 ± 54 | 74 | 185 ± 52 | 74 | 178 ± 45 | 50 |

^1^ ”By Arm” Row shows HM vitamin concentrations from all sites combined, separated by randomization arm

**Supplementary Table 3: Associations between Human Milk B-vitamin Concentrations and Infant Anthropometric Z-scores**

| Independent | LAZ-slope | WAZ-slope | WLZ-slope |
| --- | --- | --- | --- |
| Estimated Total Thiamin* | p=0.83 | p=0.51 | p=0.36 |
| Total Riboflavin (Vitamin B2) * | p=0.24 | p=0.11 | p=0.15 |
| Total Niacin (Vitamin B3) * | p=0.56 | p=0.40 | p=0.17 |
| Total vitamin B6** | p=0.17 | p=0.025  R^2^=0.025  PE= -0.00030 | P=0.042  R^2^=0.027  PE= -0.000185 |
| Total vitamin B12* | p=0.94 | p=0.48 | p=0.57 |
| Pantothenic Acid* | p=0.56 | p=0.43 | p=0.49 |
| Biotin (Vitamin B7)** | p=0.35 | p=0.70 | p=0.94 |
| Total choline | p=0.11 | p=0.29 | p=0.62 |

* = log transformed in analyses; **= square-root transformed in analyses

PE = parameter estimate for the B-vitamin variable in the model.

R^2^ and PE provided for associations with p<0.10; p<0.01 considered significant.
